# Klotho gene polymorphism, brain structure and cognition in early-life development

**DOI:** 10.1007/s11682-018-9990-1

**Published:** 2018-11-05

**Authors:** Clarisse F. de Vries, Roger T. Staff, Kimberly G. Noble, Ryan L. Muetzel, Meike W. Vernooij, Tonya White, Gordon D. Waiter, Alison D. Murray, for the Pediatric Imaging, Neurocognition and Genetics Study

**Affiliations:** 1 Aberdeen Biomedical Imaging Centre, School of Medicine, Medical Sciences and Nutrition, University of Aberdeen, Foresterhill, Aberdeen, AB25 2ZD UK; 2 Imaging Physics, Aberdeen Royal Infirmary, NHS Grampian, Foresterhill, Aberdeen, AB25 2ZD UK; 3 Teachers College, Columbia University, New York, NY 10027 USA; 4 Department of Child and Adolescent Psychiatry, Erasmus University Medical Centre, Rotterdam, Netherlands; 5 Department of Epidemiology, Erasmus University Medical Center, Rotterdam, Netherlands; 6 The Generation R Study Group, Erasmus Medical Center, Rotterdam, The Netherlands; 7 Department of Radiology and Nuclear Medicine, Erasmus University Medical Centre, Rotterdam, Netherlands

**Keywords:** Klotho, Polymorphism, Development, Cognition, Brain

## Abstract

Variation in the *klotho* gene is linked to differences in health outcomes: *klotho* allele *KL-*VS heterozygosity is associated with longevity, better cognition and greater right frontal grey matter volume in late life. Contradicting reports, however, suggest that *KL-*VS’s effect on health might be age-dependent. Here we examine the relationship between *KL-*VS genotype, cognition and brain structure in childhood and adolescence. We hypothesized that *KL-*VS has early influences on cognitive and brain development. We investigated the associations of *KL-*VS carrier status with cognition and brain morphology in a cohort of 1387 children and adolescents aged 3–21 years, examining main effects and interactions between age, sex and socioeconomic circumstance. *KL-*VS had no main effect on either cognition or brain structure, though there was a significant *KL-*VS × age interaction for cognition (specifically executive function, attention, episodic memory, and general cognition), total grey matter and total brain volume. *KL-*VS heterozygotes had better cognition than non-carriers before age 11, but lower cognition after age 11. Heterozygotes had smaller brains than non-carriers did in early childhood. Sex moderated the association between *KL-*VS and white matter volume. Among girls, *KL-*VS heterozygotes had smaller white matter volumes than non-carriers. Among boys, heterozygotes had greater white matter volumes than non-carriers. However, a replication in a cohort of 2306 children aged 6–12 years showed no significant associations. In contrast to findings in late life, these results show that *KL-*VS does not have a main effect on cognition and brain structure. Furthermore, *KL-*VS’s influence may depend on age and sex.

**Electronic supplementary material:**

The online version of this article (10.1007/s11682-018-9990-1) contains supplementary material, which is available to authorized users.

## Introduction

Children’s cognitive developmental trajectories and outcomes are highly variable, which leads to both societal and personal disparities. Lower early-life cognition has been associated with increased risk of disease and early death (Hart et al. 2003; Whalley and Deary 2001). A mixture of inherited and environmental factors lead to individual differences in childhood cognition. Increasing our understanding of the genetic and environmental causes of these developmental cognitive inequalities, and their interplay, may lead to modification strategies that maximize downstream health gains.

The *klotho* (*KL*) gene, which codes for the klotho protein, is associated with health and survival. Mice with a defective version of the gene exhibit accelerated aging (Kuro-o et al. 1997). Over-expression of klotho in mice suppressed age-related decline (Kurosu et al. 2005). In human adults, higher klotho protein levels are associated with various positive physical health outcomes, which include increased survival in lung cancer patients (Usuda et al. 2011a; Usuda et al. 2011b), and decreased risk of cardiovascular disease (Navarro-Gonzalez et al. 2014; Semba et al. 2011) and kidney function decline (Drew et al. 2017). Additionally, variation in the *KL* allele *KL-*VS is associated with differences in cognition (Dubal et al. 2014; Yokoyama et al. 2015; Mengel-From et al. 2016), brain volumes (Yokoyama et al. 2015) and survival (Arking et al. 2002; Arking et al. 2005; Invidia et al. 2010).

In adults, individuals who are heterozygous for the *KL-*VS allele outperform non-carriers on a measure of global cognition (measured as a composite of language, executive function, visuospatial function, learning and memory) (Dubal et al. 2014). Additionally, compared to non-carriers, *KL-*VS heterozygotes had greater right frontal cortical volumes and executive function, while *KL-*VS homozygotes had smaller right frontal cortical volumes and poorer executive function (Yokoyama et al. 2015). Interestingly, *KL-*VS heterozygosity has been associated with increased klotho serum levels compared to non-carriers, whereas *KL-*VS homozygosity was associated with decreased klotho levels (Yokoyama et al. 2017). However, the benefits of *KL-*VS heterozygosity may be dependent on age or environmental circumstances (Mengel-From et al. 2016; Invidia et al. 2010; de Vries et al. 2017). Conflicting reports found no difference in cognition between heterozygotes and non-carriers (Deary et al. 2005), and even a heterozygote disadvantage in cognition (Mengel-From et al. 2016). Similarly, previous work has found inconsistent associations between *KL-*VS genotype and survival (Arking et al. 2002; Arking et al. 2005; Invidia et al. 2010). Furthermore, we recently found that individuals who were heterozygous had lower white matter volumes and lower survival, but increased right frontal cortical volumes, and longitudinally, a slower cognitive decline than non-carriers (de Vries et al. 2017). This suggests that *KL-*VS heterozygosity does not necessarily have a uniformly positive effect, and can be associated with both positive and negative outcomes. Environmentally, chronic high stress may also contribute to lower levels of klotho (Prather et al. 2015). In children, socioeconomic disadvantage has been associated with markers of early-life stress (Lupien et al. 2000; Evans and Schamberg 2009), and with both early and late-life structural brain differences (Noble et al. 2015; Piccolo et al. 2016; Staff et al. 2012) and health (Luo and Waite 2005).

These conflicting reports suggest a degree of uncertainty regarding the influence of *KL-*VS heterozygosity on cognition, health and aging. Critically, the above findings are limited to studies of mice, adults and elderly individuals. The effect of *KL-*VS in childhood and its interaction with other known correlates of development, such as socioeconomic circumstance, are unknown. It is also unclear whether *KL-*VS predominantly acts on the domains of executive functioning (working memory, cognitive flexibility and inhibition), or affects other areas of cognition. More generally, it is unclear if *KL-*VS’s effect is due to differential development in childhood and/or differential aging later in life.

In this study, we investigated the associations between *KL-*VS carrier status, cognition and brain structure in 1387 children and adolescents in the Pediatric Imaging, Neurocognition and Genetics (PING) sample (Jernigan et al. 2016). Specifically, we aimed to examine the relationship between *KL-*VS carrier status and structural brain development (brain volume, cortical thickness and cortical surface area). Furthermore, we aimed to establish which domains of cognition are associated with variation in *KL-*VS. We hypothesized that *KL-*VS is associated with executive function (working memory, cognitive flexibility, and inhibition) after adjusting for possible confounders, including age, socioeconomic circumstance and genetic ancestry. We also performed exploratory analyses of the association between *KL-*VS and episodic memory, processing speed, reading, vocabulary and attention. We additionally investigated whether links with *KL-*VS are moderated by age, sex and/or socioeconomic circumstance. Finally, we investigated a possible replication of key findings in the Generation R cohort (Jaddoe et al. 2012).

## Methods

### Participants

#### PING sample

Data from the multi-site Pediatric Imaging, Neurocognition and Genetics (PING) study (Jernigan et al. 2016) were used for this study (http://ping.chd.ucsd.edu/). Participants between the ages 3 and 21 were recruited from several sites in the United States, in the areas of Baltimore, Boston, Honolulu, Los Angeles, New Haven, New York, Sacramento, and San Diego. Written informed consent was obtained from all participants or their legal guardian. The human research protections programs and institutional review boards at the universities that contributed to PING data collection and data sharing approved all experimental and consenting procedures (Brown et al. 2012).

Genome-wide genotyping was performed on DNA extracted from saliva samples using the Illumina Human660W-Quad BeadChip, from which *klotho KL-*VS genotype data were obtained (rs9536314). 1028 participants were *KL-*VS non-carriers, 347 heterozygotes, and 12 homozygotes. Due to their small numbers in the sample, *KL-*VS homozygotes were excluded from statistical analyses.

Genetic ancestry was determined by ADMIXTURE software. For each participant, the degree of African, Central Asian, East Asian, European, Native American and Oceanic ancestry was determined, resulting in six genetic ancestry factor (GAF) variables.

Household income and parental education, which were used as proxies for socioeconomic circumstance, were assessed by a questionnaire. To assess income, parents (or guardians) were asked: “What is the total income in your household from all sources over the last year?” There were 12 categories: category 1 was “< $5000 dollar”, and category 12 was “$300,000 and above”. To assess education, parents were asked “Highest Grade Completed in School”. There were 7 categories: category 1 was “Less than seven years of school” and category 7 was “Professional (MA, MS, ME, MD, PhD, LLD, and the like)”. Household income and parental education data were recoded as the means of each bin (Noble et al. 2015). The average level of parental education was used.

#### Generation R sample

The prospective population-based Generation R cohort was used as the replication sample (Kooijman et al. 2016). Parents were originally recruited to participate in this study of child and maternal health during pregnancy and have been followed ever since. When their children were between 6 and 12 years old, they visited our research center for an MRI (White et al. 2018). This sample consists of data from children who had genomic data and either had a usable scan at the age-6 assessment, or a usable scan at the age-10 assessment (total *N* = 2306, of which 1140 were girls). A majority of the sample (64%) was of Dutch ethnicity, 27% of the sample had a non-Western background, and 9% had a different (non-Dutch) Western background. All procedures were approved by the Medical Ethical Committee at the Erasmus MC University Medical Center, Rotterdam, and participants provided informed consent/assent.

The generation and management of GWAS genotype data for the Generation R Study was performed at the Genetic Laboratory of the Department of Internal Medicine, Erasmus MC, the Netherlands. DNA from whole blood at birth was analyzed using Illumina 610 K/660 W. Filtering for sample (≥97.5%) and SNP call rates (≥95%), minor allele frequency ≥ 1% and deviations from Hardy-Weinberg equilibrium (*p* < 10–7) were conducted (https://www.ncbi.nlm.nih.gov/pubmed/25762173). 1698 participants were *KL-*VS non-carriers, and 608 were heterozygotes. Data were imputed with the Haplotype Reference Consortium v1.1 panel (https://imputationserver.sph.umich.edu/index.html). Twenty principal components of ancestry (PCA) were calculated for the whole Generation R sample (*N* = 5731).

### Image acquisition and processing

#### PING sample

Magnetic resonance imaging (MRI) scans (T1-weighted, T2-weighted, diffusion-weighted, and resting-state functional MRI) were collected using 12 3 Tesla scanners. The T1-weighted scans were processed and segmented using an adapted version of the FreeSurfer software package to obtain measures of brain volumes, cortical thickness and cortical surface area (Jernigan et al. 2016).

Measures of total grey matter volume, total white matter volume, total brain volume and total intracranial volume were obtained using the SPM software package (SPM12, version 6470) (Ashburner 2009). We opted to derive these measures with SPM12, because it has previously been demonstrated that SPM12’s estimate of total intracranial volume outperforms FreeSurfer’s segmentation in a pediatric sample (Sargolzaei et al. 2015). SPM12’s segmentation algorithm was employed in conjunction with customized pediatric tissue probability maps. These tissue probability maps were generated with SPM8, using the Template-O-Matic Toolbox (Wilke et al. 2008), and are based on the sex and mean age of the imaged participants.

The segmented grey and white matter probability maps were then normalized to MNI space using a sample-specific DARTEL template (Ashburner 2007), and smoothed with an 8 mm kernel.

#### Generation R sample

Structural imaging data were collected on 3 Tesla MRI systems (GE, MR750, *N* = 309; MR750W, *N* = 1997), Milwaukee, WI, Muetzel et al. 2017). Images were processed using the FreeSurfer image analysis suite (version 6.0) to obtain estimates of intracranial volume, total brain volume, total gray matter volume and total white matter volume (Fischl 2012). Surface reconstructions were visually inspected for accuracy, and data not suitable for analysis were excluded leaving 2306 datasets with usable imaging and genomic data (Muetzel et al. 2017).

### Cognitive testing

#### PING sample

The cognition of PING participants was assessed by administering the NIH Toolbox Cognition Battery (NTCB) (Gershon et al. 2013; Akshoomoff et al. 2014). The NTCB consists of seven cognitive tests, which produced eight cognitive scores.

The Dimensional Change Card Sort Test measures cognitive flexibility and attention. Participants are presented with images that vary along two dimensions (shape and color), and were asked to match one of the two images with a target image along one of the two dimensions.

The Picture Sequence Memory Test measures episodic memory. Participants are shown a sequence of images. They are then presented with those same images, and are asked to place them in the correct order.

The List Sorting Working Memory Test measures working memory. Participants are presented with a series of images together with the name of what is depicted in the image. They are then asked to recall and sort them according to size (smallest to largest).

The Pattern Comparison Processing Speed Test measures speed of processing. Participants are shown two images, side by side, and are asked whether or not they are the same. The test score was given by the total number of correct responses within the time limit of 90 s.

The Picture Vocabulary Test assesses receptive vocabulary. Participants are presented with a sound recording of a word, and are shown four images. They are asked to match the sound with the image that most closely corresponds to the meaning of the word.

For the Oral Reading Recognition Test participants are asked to read a series of letters and words aloud. Depending on age, between 70 and 125 items were administered. The test score was given by the total number of correct responses.

The Flanker Inhibitory Control and Attention Test assesses attention and inhibitory control. Participants were asked to indicate whether an object was pointing to the left or right. The object was flanked by other objects that pointed either in the same direction (congruent), or in the opposite direction (incongruent). The flanker task resulted in two scores: an inhibition score based on both the congruent and incongruent responses, and an attention score based only on the congruent responses.

#### Generation R sample

For a subset of the 2306 children with imaging and genomic data, 2302 children (1493 *KL-*VS non-carriers, 539 heterozygotes) were assessed at the age-6 assessment using an abbreviated version of the Snijders-Oomen Niet-verbale Intelligentie Test – Revisie (SON-R 2½-7) (Tellegen et al. 2005; Tiemeier et al. 2012). A non-verbal intelligence quotient was estimated from the two SON-R performance subtests that were administered (*Mosaics* and *Categories*), which is highly correlated with estimates resulting from the complete version (Basten et al. 2014).

### Statistical analysis

T-tests and general linear modeling were performed using SPSS version 24. Initially, associations were examined in the PING cohort. First, t-tests examined whether there were differences between heterozygotes and non-carriers in genetic ancestry, age at cognitive testing, household income, parental education, and any of the cognitive tests and global brain measures (*p* < .05). Then, general linear models were employed to examine differences in cognition and global brain metrics. Differences in regional brain metrics were analyzed using the PING study data portal (Bartsch et al. 2014) and voxel-based morphometry (VBM). Subsequently, it was examined whether key associations could be replicated in the Generation R cohort.

#### PING - cognition

Guided by previous work in adults in late life (Yokoyama et al. 2015), we examined whether there was a difference between *KL-*VS heterozygotes and non-carriers in measures of executive function: working memory (List Sorting Working Memory), cognitive flexibility (Dimensional Change Card Sort), and inhibition (Flanker Inhibitory Control score). As an exploratory analysis, we examined whether *KL-*VS genotype was associated with differences in the Picture Sequence Memory Test (episodic memory), Flanker attention score, the Pattern Comparison Processing Speed Test, Picture Vocabulary Test, and Reading Recognition Test.

We examined whether there were interactions between *KL-*VS and sex, age, age^2^, household income and parental education. Both a linear and quadratic age term were included in order to model the expected curvilinear relationship between age and cognition. Age was standardized prior to calculating age^2^ in order to reduce the multicollinearity between these terms and their interactions. As income was positively skewed, the natural logarithm of income was included in the models. Covariates included sex, genetic ancestry factors (GAF), age, age^2^, household income, and parental education.

We examined whether *KL-*VS had a differential effect on any specific cognitive domain, by pairwise comparing the beta-values. We standardized the cognitive scores, and computed the Z-scores for each comparison by subtracting the two betas from each other and dividing it by the square root of the sum of their squared error terms. There was a significant difference in betas when the Z-score’s absolute value is larger than 1.96.

#### PING - brain imaging

We examined whether there were differences between *KL-*VS heterozygotes and non-carriers in brain MRI derived total brain volume, total grey matter volume and total white matter volume (correcting for total intracranial volume). We also examined whether there were differences in mean cortical thickness, and total brain surface area, between *KL-*VS heterozygotes and non-carriers. We examined whether there were interactions between *KL-*VS and sex, age, age^2^, household income and parental education. Again, age was modeled with a linear and quadratic term, and income with a logarithmic term. We corrected for sex, genetic ancestry factors (GAF), age, age^2^, household income, parental education, and MRI scanner.

Regional brain analysis was performed in two ways. The PING study data portal (Bartsch et al., 2014) was used to examine local differences in cortical surface area, thickness and volume. As many regions are compared in this type of unbiased whole brain analysis, False Discovery Rate (FDR) multiple comparison correction was employed. We examined whether there were interactions between *KL-*VS and sex, age, age^2^, household income and parental education. Covariates included sex, genetic ancestry factors (GAF), age, age^2^, household income, parental education, and MRI scanner (dummy-coded). Regional cortical volume was additionally corrected for total brain volume.

Furthermore, using SPM12, VBM was performed on the normalized and smoothed tissue probability maps. Local differences in grey and white matter volume were examined voxel-by-voxel in an unbiased whole brain analysis. In addition, small volume correction was used to examine differences in right dorsolateral prefrontal cortex (rDLPFC) volume, which was previously found to be associated with *KL-*VS genotype (Yokoyama et al. 2015). Family-wise error (FWE) multiple comparison correction was employed, with a minimum cluster extent of 5 voxels. The final model that was applied to the global brain volume variables was employed, correcting for total brain volume instead of total intracranial volume.

#### Generation R replication

The following FreeSurfer segmentations were used as outcomes in the Generation R general linear models: total brain volume (supratentorium (no ventricles) plus cerebellum), total gray matter and total white matter volumes (both from surface-based estimates). Models were adjusted for age at MRI, age^2^, sex, total intracranial volume, genetic ancestral background (first 7 principle components), and MRI scanner, with *klotho* status as the predictor. In addition, it was examined whether age-6 general intellectual ability was associated with *KL-*VS carrier status*.* The model was adjusted for age at cognitive testing, age^2^, sex, genetic ancestral background, income and maternal education.

First, a main effect of *KL-*VS was tested. Subsequently, in separate models, the two-way interaction effects of age-by-*klotho* and sex-by-*klotho* were tested.

## Results

### Cognition

Table 1 shows a demographic description of the PING sample, split by *KL-*VS non-carriers, heterozygotes and homozygotes. Relative to non-carriers, heterozygotes have significantly higher proportions of European (t(652.42) = 5.43, *p* < .001) and African (t(521.54) = 2.62, *p* = .009) ancestry, and lower proportions of Native American (t(693.98) = −3.28, *p* = .001), East Asian (t(1289.86) = −10.74, p < .001) and Oceanic ancestry (t(1196.00) = −4.33, p < .001). There was no difference in proportion of Central Asian ancestry (t(1373) = 0.32, *p* = .747). The participants were grouped in ancestry groups, corresponding to >50% of any ancestry; the remaining participants were categorized in the group Mixed (see Table 1). The frequency of *KL-*VS heterozygosity varies across ancestry groups. Notably, the East Asian ancestry group has the lowest frequency of *KL-*VS heterozygosity (7%). Previous work has found that the *KL-*VS *klotho* variant did not exist in Korean and Japanese populations (Kim and Jeong 2016). Investigating whether *KL-*VS heterozygosity appears in the East Asian ancestry group because of mixed heritage, we varied the threshold for group membership from >50% to >85% in 5% increments. The frequency of heterozygosity decreases until it reaches 0% at >85% ancestry (*N* = 109). There were no differences in age at cognitive testing, household income, parental education, or any of the cognitive tests or brain measures.

**Table 1:** Demographic description of the PING sample

	Non-carriers *N* = 1028	Heterozygotes *N* = 347	Homozygotes *N* = 12
Sex-Number Male	508, 49.6%	195, 56.9%	9, 75.0%
Age at Cognitive Testing	11.69 (0.16)	11.71 (0.25)	12.26 (1.59)
Household Income	$97,411 (2439)	$98,853 (4507)	$99,167 (27,865)
Parental Education (years)	14.96 (0.07)	15.10 (0.13)	15.58 (0.63)
Genetic ancestry factor (GAF)			
European	.599 (.012)	.717† (.018)	.806 (.099)
African	.124 (.008)	.172† (.016)	.184 (.101)
Native American	.055 (.004)	.033† (.006)	.011 (.008)
East Asian	.188 (.010)	.047† (.008)	.000 (.000)
Oceanic	.009 (.001)	.003† (.001)	.000 (.000)
Central Asian	.025 (.004)	.027 (.007)	.000 (.000)
> 50% European ancestry (N)	607	248 [28.7%]	10
> 50% African ancestry (N)	116	64 [35.2%]	2
> 50% Native American ancestry (N)	11	5 [31.3%]	0
> 50% East Asian ancestry (N)	187	14 [7.0%]	0
> 50% Oceanic ancestry (N)	0	0	0
> 50% Central Asian ancestry (N)	18	6 [25%]	0
Mixed ancestry (N)	89	10 [10.1%]	0
Cognitive test scores			
Picture Sequence Memory	25.43 (0.37)	25.53 (0.58)	28.50 (3.62)
List sorting	17.29 (0.18)	17.75 (0.27)	20.00 (1.50)
Pattern Comparison	36.43 (0.38)	36.58 (0.60)	36.67 (2.88)
Picture Vocabulary	0.58 (0.05)	0.69 (0.07)	1.09 (0.43)
Oral Reading	122.4 (2.2)	123.7 (3.6)	145.5 (22.7)
Flanker Inhibition	7.56 (0.06)	7.65 (0.09)	7.52 (0.60)
Flanker Attention	8.03 (0.06)	8.13 (0.09)	7.92 (0.59)
Dimensional Change Card Sort	7.61 (0.05)	7.71 (0.08)	7.46 (0.46)
*g*	−0.007 (0.035)	0.019 (0.051)	0.017 (0.345)
Cortical measures	*N* = 877	*N* = 306	N = 12
Mean Cortical Thickness [mm]	2.784 (0.006)	2.784 (0.009)	2.806 (0.054)
Total Cortical Surface Area [mm^2^ × 10^3^]	170.4 (0.6)	171.0 (1.0)	175.6 (7.5)
Brain volumes	*N* = 636	*N* = 251	N = 10
Total Grey Matter Volume [mm^3^ × 10^6^]	0.737 (0.003)	0.747 (0.005)	0.753 (0.016)
Total White Matter Volume [mm^3^ × 10^6^]	0.425 (0.002)	0.427 (0.004)	0.439 (0.020)
Total Brain Volume [mm^3^ × 10^6^]	1.162 (0.005)	1.174 (0.008)	1.192 (0.034)
Total Intracranial Volume [mm^3^ × 10^6^]	1.360 (0.005)	1.378 (0.009)	1.408 (0.049)

Mean values are shown. The numbers in round brackets indicate the standard error. The numbers in square brackets indicate the frequency of *KL*-VS heterozygosity in each ancestry group. N indicates the maximum number of participants used in analyses. † indicates a significant difference between heterozygotes and non-carriers at *p* < .01 level

As performance on the eight cognitive scores was expected to be highly correlated, we performed a principal component analysis and extracted a single, unrotated principal component from the data. This principal component can be a considered as a measure of general cognitive ability (*g*) (Spearman 1904; Jensen 1998). Here *g* accounted for 73.2% of the overall variability across the eight cognitive scores. The component loadings were: .788 for Picture Sequence Memory (which measures episodic memory), .856 for List Sorting (working memory), .814 for Pattern Comparison (processing speed), .854 for Picture Vocabulary (receptive vocabulary), .879 for Oral Reading (reading recognition), .905 and .857 for Flanker (inhibition and attention), and .887 for Dimensional Change Card Sort (cognitive flexibility).

Models were constructed to examine whether *KL-*VS carrier status was associated with cognition and brain measures, when adjusting for covariates. As income and education are highly correlated with each other, models considered education and income effects separately. The initial models comprised the terms *KL-*VS, sex, GAF, age, age^2^, education (or income), and *KL-*VS × education (or *KL-*VS × income). The interaction terms were not significant (*p* > .05) and were therefore dropped from the models. Then, interactions of *KL-*VS with sex and age were examined in a model with the terms: *KL-*VS, sex, education, income, GAF, age, age^2^, *KL-*VS × sex, *KL-*VS × age, and *KL-*VS × age^2^. Non-significant interaction terms were removed in an iterative process, examining higher-order interactions first. *KL-*VS × age^2^ and *KL-*VS × sex were not significant and were removed from the models.

Table 2 shows the results from the final general linear models. *KL-*VS carrier status had no main effect for any of the cognitive test scores. However, the *KL-*VS × age interaction was significantly associated with certain cognitive test scores. Specifically, the association between *KL-*VS and executive function varied by age, with a significant *KL-*VS × age interaction for inhibition (*p* = .015) and cognitive flexibility (*p* = .001), but not for working memory (*p* = .116). In exploratory analyses, a significant *KL-*VS × age interaction was found for episodic memory (*p* = .002) and attention (*p* = .022). No *KL-*VS x age interactions were found for processing speed (*p* = .642), vocabulary (*p* = .208) and reading (*p* = .699). For the composite cognitive score *g*, while there was no *KL-*VS main effect, a *KL-*VS × age interaction was found (*p* < .001).

**Table 2:** PING sample estimated coefficients of the general linear models for the cognitive scores

	Picture sequence memory	List sorting	Pattern comparison	Picture vocabulary	Oral reading	Flanker inhibition	Flanker attention	Dimensional change card sort	*g*
	episodic memory	working memory	processing speed	receptive vocabulary	reading recognition	inhibitory control	attention	cognitive flexibility	general cognition
Intercept	−0.569* (0.232)	−0.448* (0.205)	−0.528* (0.224)	−1.139† (0.181)	−0.746† (0.171)	−0.140 (0.212)	−0.084 (0.242)	−0.440* (0.223)	−0.782† (0.154)
*KL*-VS	0.028 (0.041)	−0.003 (0.036)	0.016 (0.040)	−0.026 (0.032)	−0.017 (0.030)	−0.005 (0.038)	−0.028 (0.043)	−0.054 (0.040)	−0.023 (0.027)
Sex	0.016 (0.035)	−0.078* (0.031)	0.089† (0.034)	−0.039 (0.027)	−0.029 (0.026)	−0.015 (0.032)	−0.004 (0.037)	0.120† (0.034)	0.037 (0.023)
Age	0.690† (0.037)	0.774† (0.033)	0.807† (0.036)	0.820† (0.029)	0.907† (0.028)	0.754† (0.034)	0.669† (0.039)	0.785† (0.037)	0.951† (0.026)
Age^2^	−0.319† (0.019)	−0.427† (0.017)	−0.254† (0.019)	−0.190† (0.015)	−0.187† (0.014)	−0.472† (0.018)	−0.471† (0.020)	−0.418† (0.020)	−0.404† (0.014)
Income	0.045* (0.022)	0.043* (0.020)	0.037 (0.022)	0.069† (0.017)	0.039* (0.016)	0.015 (0.020)	0.015 (0.023)	0.033 (0.021)	0.045† (0.015)
Education	0.025† (0.010)	0.032† (0.009)	0.022* (0.009)	0.048† (0.008)	0.036† (0.007)	0.029† (0.009)	0.026† (0.010)	0.024* (0.009)	0.037† (0.006)
GAF African	−0.414† (0.073)	−0.323† (0.064	−0.139* (0.071)	−0.558† (0.057)	−0.319† (0.054)	−0.099 (0.066)	−0.113 (0.076)	−0.160* (0.070)	−0.345† (0.048)
GAF Native American	0.286 (0.163)	−0.132 (0.145)	−0.341* (0.158)	−0.552† (0.128)	−0.090 (0.119)	−0.168 (0.149)	−0.103 (0.170)	−0.298 (0.155)	−0.200 (0.106)
GAF East Asian	0.076 (0.068)	−0.073 (0.060)	0.052 (0.066)	−0.085 (0.053)	0.069 (0.050)	0.121 (0.062)	0.194† (0.071)	0.116 (0.065)	0.093* (0.045)
GAF Oceanic	0.026 (0.658)	−0.620 (0.588)	0.974 (0.638)	−1.943† (0.515)	−0.691 (0.494)	−0.863 (0.618)	−1.971† (0.704)	−1.441* (0.656)	−1.180† (0.449)
GAF Central Asian	−0.041 (0.152)	0.057 (0.133)	−0.175 (0.147)	−0.004 (0.119)	−0.126 (0.111)	0.118 (0.138)	0.210 (0.157)	0.036 (0.140)	−0.017 (0.095)
*KL*-VS × Age	0.129† (0.042)	0.059 (0.038)	0.019 (0.041)	0.042 (0.033)	0.012 (0.031)	0.094* (0.039)	0.101* (0.044)	0.138† (0.042)	0.105† (0.029)

† indicates terms significant at *p* < .01 level. * denotes terms significant at *p* < .05 level. The numbers inside the brackets indicate the standard errors. GAF stands for genetic ancestry factor; GAF European was chosen as the reference group. N ranges from 1079 to 1242

**Fig. 1:**
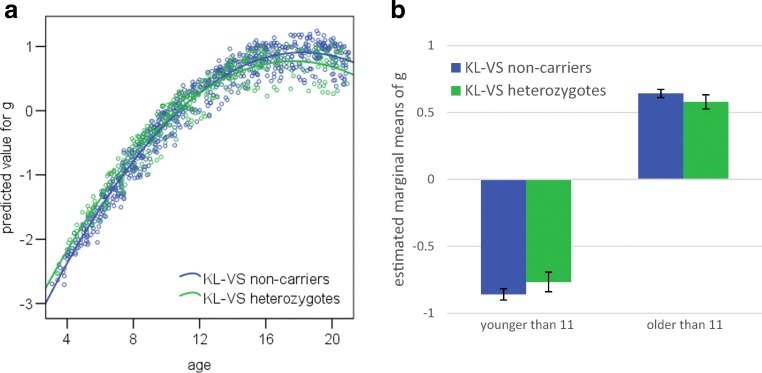
In the PING sample, there was a significant *KL-*VS × age interaction for cognition. (**a**) Predicted value for *g* versus age scatter plot. *N* = 1079. (**b**) Estimated marginal means of *g*, split by age and *KL-*VS genotype. The error bars represent the 95% confidence interval. N_age_ < 11 = 479, N_age_ > 11 = 600

Fig. 1a depicts the significant *KL-*VS × age interaction for *g*. In early to middle childhood, heterozygotes appear to slightly outperform non-carriers. Thereafter, however, heterozygotes show a less steep increase in cognition with age. Dividing the cohort into two groups (younger and older than 11 years, or the approximate start of puberty) and examining the raw data, we find that heterozygotes perform about 0.23 standard deviations ahead of their non-carrier peers on global cognition in early to middle childhood. However, by mid- to late-adolescence, heterozygotes perform about 0.25 standard deviations behind non-carriers on global cognition. To probe these differences further, the same general linear models that were used to examine associations with cognition for the whole cohort (see Table 2), are applied to the two groups (younger and older than 11 years), with the *KL-*VS × age interaction removed. Table 3 shows the difference of the estimated marginal means of the standardized cognitive scores between heterozygotes and non-carriers, and the corresponding *p*-values. Younger heterozygotes performed slightly but significantly better than non-carriers did on cognitive flexibility and *g*, whereas older heterozygotes performed slightly but significantly worse than non-carriers did on inhibition, attention and *g* (see Fig. 1b for estimated marginal means of g, split by age and *KL-*VS genotype).

**Table 3:** PING sample comparison of cognitive scores between *KL-*VS heterozygotes and non-carriers, split by age

	Below age 11		Above age 11	
	*Δ*	*p*	*Δ*	*p*
Picture Sequence Memory	0.034	.507	−0.114	.076
List Sorting	0.005	.921	−0.013	.777
Pattern Comparison	0.038	.465	−0.063	.293
Picture Vocabulary	0.026	.507	0.024	.644
Oral Reading	0.002	.950	0.008	.874
Flanker Inhibition	0.080	.215	**−0.079**	**.003**
Flanker Attention	0.120	.099	**−0.066**	**.016**
Dimensional Change Card Sort	**0.163**	**.021**	−0.060	.122
*g*	**0.093**	**.034**	**−0.063**	**.045**

Δ indicates the difference of the estimated marginal means of the standardized cognitive scores between* KL*-VS heterozygotes and non-carriers. *p* indicates the corresponding *p*-values. A negative sign (−) indicates that heterozygotes have lower means than non-carriers do. Bold signifies *p* < .05. N_age < 11_ ranges from 479 to 628; N_age > 11_ ranges from 600 to 614

We then compared the effect sizes of the *KL-*VS × age interaction across the eight cognitive scores. The effect size for episodic memory (measured by Picture Sequence Memory, 0.129) was significantly higher than that for reading (Oral Reading, 0.012). The effect size for cognitive flexibility (Dimensional Change Card Sort, 0.138) was significantly higher than those for reading (0.012) and processing speed (Pattern Comparison, 0.019). However, these differences do not withstand multiple comparison correction, and there were no other significant differences in effect size, justifying the use of *g* as a summary measure of general cognitive ability in this cohort.

In order to examine whether genetic ancestry could influence the found associations with cognition, the analysis was restricted to participants with >50% European ancestry (*N* = 677). A significant *KL-*VS × age interaction was found for *g* (*p* = .006), in the same direction as seen before: younger heterozygotes outperform their non-carrier peers, while older heterozygotes perform worse than non-carriers did. Splitting the group by age (as before), shows that this differences is significant in children younger than 11 years (*p* = .017), but not in children older than 11 (*p* = .688). Investigating further by restricting the analysis to participants with >50% African ancestry (*N* = 139) we again found a significant *KL-*VS × age interaction for *g* (*p* = .034) in the same direction. Splitting the group by age now shows a significant difference in cognition for children older than 11 (*p* = .019), but not for children younger (*p* = .939).

The replication in the Generation R cohort showed no significant main effect of *KL-*VS for non-verbal IQ at 6 years-of-age (*p* = .327), and no significant *KL-*VS × age interaction (*p* = .173).

### Brain analysis

We first examined whether *KL-*VS carrier status was associated with total grey matter volume, total white matter volume or total brain volume. In preliminary models, terms that were not significant for total grey/white/brain volume were removed from the models in an iterative process; higher-order interactions were examined first. Interactions of *KL-*VS with income and education were again examined in two separate models. The models comprised the terms *KL-*VS, sex, age, age^2^, GAF, scanner, total intracranial volume, education (or income), and *KL-*VS × education (or *KL-*VS × income). Income, education, *KL-*VS × education, and *KL-*VS × income were not significant. Next, interactions of *KL-*VS with sex and age were examined in a model consisting of the terms: *KL-*VS, sex, age, age^2^, GAF, scanner, total intracranial volume, *KL-*VS × sex, *KL-*VS × age, and *KL-*VS × age^2^. *KL-*VS × age^2^ was not significant and was removed from the models.

**Fig. 2:**
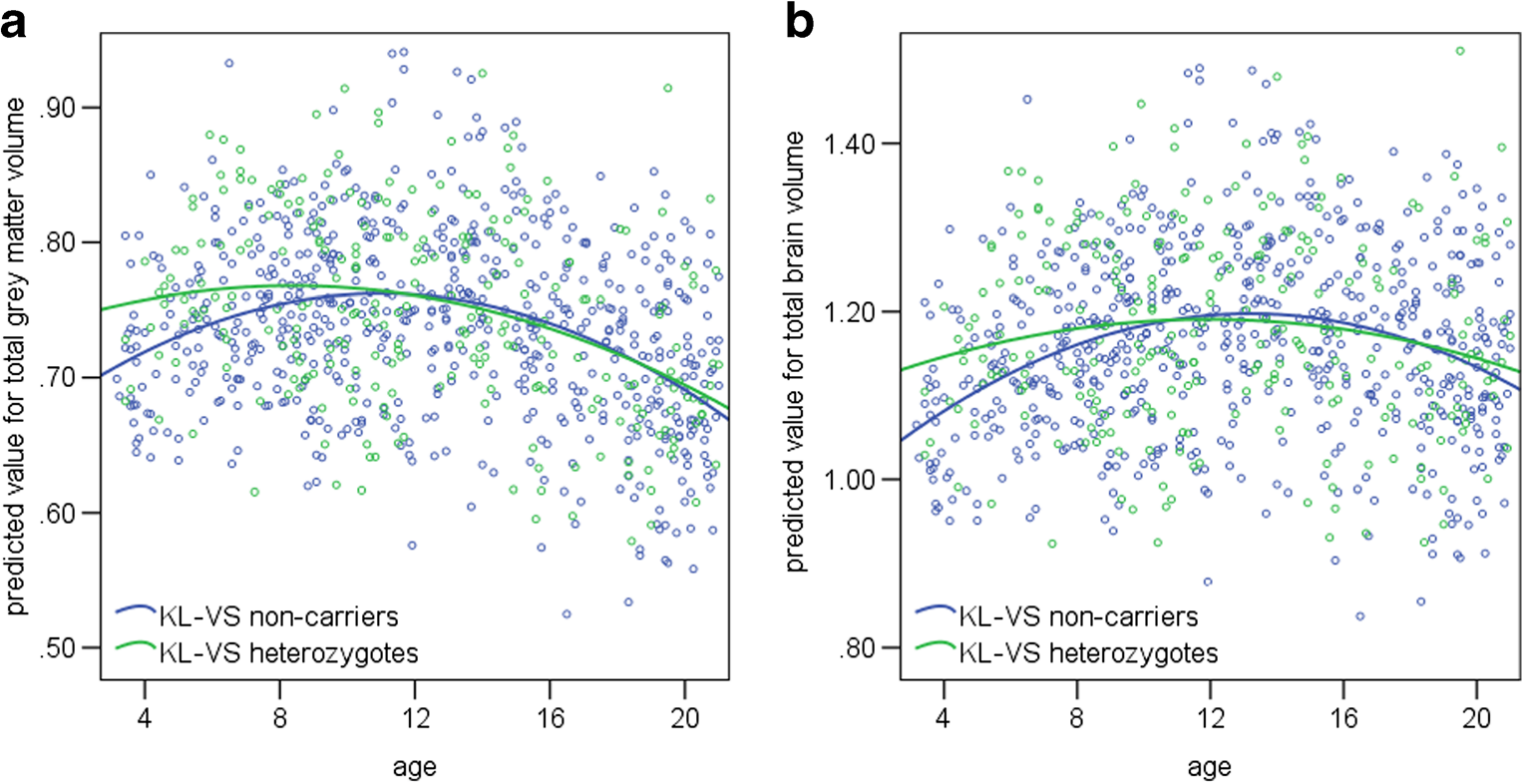
The PING sample showed a significant *KL-*VS × age interaction for brain volume. Scatter plots of age versus predicted value for total grey matter volume (**a**), and total brain volume (**b**) [mm^*3*^ × 10^6^]. *N* = 872

Table 4 shows the results from the final general linear models. *KL-*VS had no main effect. The *KL-*VS × age interaction was significantly associated with total brain volume (*p* = .039) and total grey matter volume (*p* = .045), but not total white matter volume (*p* = .559). Figure 2 shows plots of age versus the total grey matter/total brain volume value predicted by the general linear model, split by *KL-*VS heterozygotes and non-carriers. The figure and the significant *KL-*VS × age interaction suggest that heterozygotes have less total grey matter and total brain volume than non-carriers do in early childhood, but catch up when older, and subsequently show similar rates of decline.

**Table 4:** PING sample estimated coefficients of the general linear models for total grey/white/brain volume

	Total Brain Volume	Total Grey Matter Volume	Total White Matter volume
Intercept	0.115† (0.019)	0.121† (0.016)	−0.006 (0.014)
*KL*-VS	−0.002 (0.004)	0.001 (0.003)	−0.002 (0.003)
Sex	−0.009 (0.005)	0.005 (0.004)	−0.014† (0.004)
Age	−0.027† (0.003)	−0.034† (0.002)	0.007† (0.002)
Age^2^	−0.006† (0.001)	−0.005† (0.001)	−0.001 (0.001)
GAF African	−0.031† (0.005)	−0.030† (0.004)	−0.002 (0.004)
GAF Native American	−0.007 (0.013)	−0.018 (0.010)	0.011 (0.009)
GAF East Asian	7E-05 (0.006)	−0.004 (0.005)	0.004 (0.004)
GAF Oceanic	−0.118 (0.250)	−0.154 (0.203)	0.035 (0.182)
GAF Central Asian	−0.004 (0.009)	−0.003 (0.007)	−0.001 (0.006)
Scanner 1	−0.091† (0.005)	−0.056† (0.004)	−0.036† (0.004)
Scanner 2	0.000 (0.005)	−0.003 (0.004)	0.003 (0.004)
Scanner 3	0.001 (0.005)	0.006 (0.004)	−0.005 (0.004)
Scanner 4	−0.014† (0.005)	−0.011† (0.004)	−0.003 (0.004)
Scanner 5	−0.085† (0.006)	−0.050† (0.005)	−0.035† (0.004)
Scanner 6	−0.014† (0.005)	−0.008 (0.004)	−0.006 (0.004)
Scanner 7	0.004 (0.005)	0.007 (0.004)	−0.003 (0.004)
Scanner 8	0.005 (0.011)	0.004 (0.009)	0.001 (0.008)
Total intracranial volume	0.799† (0.012)	0.473† (0.010)	0.326† (0.009)
*KL*-VS × Age	0.006* (0.003)	0.005* (0.002)	0.001 (0.002)
*KL*-VS × Sex	0.004 (0.006)	−0.004 (0.005)	0.008* (0.004)

† indicates terms significant at *p* < .01 level. * denotes terms significant at *p* < .05 level. The numbers inside the brackets indicate the standard errors. GAF stands for genetic ancestry factor; GAF European was chosen as the reference group. N = 872

**Fig. 3:**
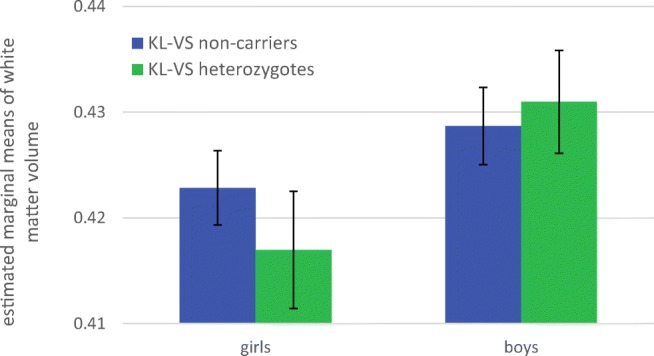
Sex moderated the association between *KL-*VS and white matter volume in the PING sample. Estimated marginal means of total white matter volume [mm^*3*^ × 10^6^], split by sex and *KL-*VS genotype. The error bars represent the 95% confidence interval. N_girls_ = 427, N_boys_ = 445

*KL-*VS × sex was significantly associated with total white matter volume (*p* = .049), but not total grey matter (*p* = .361) or total brain volume (*p* = .492). Figure 3 shows the estimated marginal means of total white matter volume, split by sex and *KL-*VS genotype. Among girls, *KL-*VS heterozygotes had smaller total white matter volumes than non-carriers, whereas among boys, heterozygotes had greater white matter volumes than non-carriers. An analysis of the simple effects shows no significant difference in total white matter volume between heterozygotes and non-carriers for either girls (*p* = .059) or boys (*p* = .409).

Limiting the analysis to participants with >50% European ancestry (*N* = 614) found that *KL-*VS × sex was not significant for total white matter volume (*p* = .230). A further analysis using participants with >50% African ancestry (*N* = 122), however, did find a significant *KL-*VS × sex interaction (*p* = .023) in the same direction as seen before. The simple effects analysis again shows no significant difference between heterozygotes and non-carriers, for girls (*p* = .136) or for boys (*p* = .073). In both ancestry groups, *KL-*VS × age was not significant for total brain volume (p_European_ = .129; p_African_ = .950) or total grey matter volume (p_European_ = .160; p_African_ = .471).

The above global brain volume findings did not pass a multiple testing threshold. There were no significant differences (*p* > .05) between *KL-*VS heterozygotes and non-carriers in mean cortical thickness and total cortical surface area. The interaction terms *KL-*VS × income, *KL-*VS × education, *KL-*VS × sex, *KL-*VS × age, and *KL-*VS × age^2^ were also not significant. There were also no significant differences (p > .05, FDR corrected) in regional cortical thickness, surface area, and volume. The interaction terms *KL-*VS × income, *KL-*VS × education, *KL-*VS × sex, *KL-*VS × age, and *KL-*VS × age^2^ were again not significant. In addition, the VBM analysis showed no significant differences in regional grey or white matter volume, or rDLPFC volume (p > .05, FWE corrected).

In the Generation R cohort replication (see Supplementary Table 1), there was no main effect of *KL-*VS for total brain volume, total grey matter volume, and total white matter volume. The interaction terms *KL-*VS × sex and *KL-*VS × age were also not significant.

## Discussion

In both the PING sample of 1387 children and adolescents, and the Generation R sample of 2306 children studied here, we found that *klotho* allele *KL-*VS has no main effect on cognition or brain structure. These results suggest that the differences previously described in late life may be acquired throughout the life-course, as opposed to laid down in childhood. Furthermore, findings in the PING sample suggest that *KL-*VS’s influence may depend on age and sex.

In the PING sample, *KL-*VS is associated with different trajectories of brain and cognitive development in childhood and adolescence. Specifically, *KL-*VS heterozygotes had an advantage in cognition in early to middle childhood, but a disadvantage through adolescence. *KL-*VS heterozygotes also had larger brains in early-to-middle childhood; this neurodevelopmental difference was not sustained in adolescence. Associations between *KL-*VS and cognitive and brain outcomes did not vary by socioeconomic circumstance (measured by parental education or household income), which has previously been shown to influence cortical surface area in this sample (Noble et al. 2015). The subgroup analyses show that genetic ancestry and population substructure influence the found associations. For the cognitive findings, while the influence of *KL-*VS varies with age in both the African and European ancestry group, the European group appears to drive the heterozygote advantage in younger children, while the African group appears to drive the heterozygote disadvantage in older children. For the brain findings, no interaction with age was seen for either ancestry group.

The cognitive analyses in PING suggest that *KL-*VS is associated with executive functioning and with general cognitive ability, supporting previous work in older adults (Dubal et al. 2014; Yokoyama et al. 2015). Exploratory analyses indicate that *KL-*VS is also associated with episodic memory and attention. Further, non-carriers have greater age-related increases in cognition than do heterozygotes. This interaction supports the previously proposed possibility of an age-dependent effect of *KL* in older adults (Mengel-From et al. 2016; de Vries et al. 2017). Previous contradictory reports on the association between *KL-*VS carrier status and cognition may be explained by differences in the age range of the samples and/or the measures of cognition that were examined.

In the PING sample, the association between *KL-*VS carrier status and white matter volume varied by sex. Non-carrier girls tended to have greater white matter volume than heterozygote girls, whereas heterozygote boys tended to have greater white matter volume than non-carrier boys (though post-hoc analyses found no significant difference in total white matter volume between heterozygotes and non-carriers within either sex). The subgroup analysis suggests that this interaction may be driven by participants with a majority of African ancestry. It is possible that (1) differential development in early life might partially explain the previously found lower total white matter volumes for heterozygotes in late life (de Vries et al. 2017), and (2) heterozygosity in women might be driving this disadvantage. However, the lower white matter volumes for heterozygotes in late life were found in a homogeneous Scottish cohort. Of note, lower total white matter volumes in late life are associated with decreased longevity (Van Elderen et al. 2016).

Sex- and age-related trajectories in neurodevelopment are well-established, and support the found *KL-*VS interactions with sex and age. Boys have more global white matter volume than girls (Wilke et al. 2007; Lenroot and Giedd 2006); this difference is sustained in adults (Gur et al. 1999; Chen et al. 2007; Good et al. 2001). Boys and girls also follow different trajectories of white matter growth during development (De Bellis et al. 2001; Giedd et al. 1999). In addition, total grey matter volume follows an inverted U-shape with increasing age (Giedd and Rapoport 2010). The initial increase in volume may relate to dendritic arborisation (Giedd 2004), while the subsequent decrease likely reflects dendritic pruning processes (De Bellis et al. 2001). Previous work in mice has shown that klotho plays a role in neurodevelopment, including myelination (Chen et al. 2013), synaptic function (Dubal et al. 2014) and dendritic arborization (Laszczyk et al. 2017).

There were no significant differences in mean or regional cortical thickness, total or regional cortical surface area or regional grey or white matter volume between *KL-*VS heterozygotes and non-carriers. Specifically, heterozygotes’ right dorsolateral prefrontal cortex (rDLPFC) volume advantage seen in late life (Yokoyama et al. 2015) was not observed in this early-life sample. This suggests that differences in trajectories of aging, not development, may cause heterozygotes’ greater rDLPFC volume seen in late life.

*KL-*VS homozygotes were not considered in the statistical analysis because of their small numbers (*N*_*PING*_ = 12). Interestingly, however, for five out of the eight administered cognitive tests (Picture Sequence Memory, List Sorting, Pattern Comparison, Picture Vocabulary, Oral reading) homozygotes outperformed both heterozygotes and non-carriers. This is unexpected, as previous reports have suggested that *KL-*VS homozygosity has detrimental effects on cognition and right frontal brain volumes. One possibility is that the detrimental effects of *KL-*VS homozygosity might not be uniformly present at all stages in life, and perhaps even have positive effects early in development.

Crucially, none of the interactions found in the PING sample were replicated in the larger Generation R sample. The non-replication, in addition to the modest *p*-values that do not pass a multiple testing threshold, suggest that the found interactions might be false positives. Furthermore, the subgroup analysis indicates that population substructure has an influence on these associations. However, as this is only one study in a body of literature, and one of the few that examines the influence of *klotho* genotype in children, future work should continue to explore these interactions. The different age-distribution/narrower age-range of the Generation R sample (6 to 12 years, compared to 3 to 21 years) could have contributed to the lack of replication. Moreover, participants of the two cohorts were administered different tests of cognition, and our analysis in the PING sample suggests that *KL-*VS is associated with specific cognitive domains.

These results complicate a “straightforward” interpretation of the influence of *KL-*VS heterozygosity as beneficial. We find no main effect of *KL-*VS genotype. In addition, interactions with age, sex and ethnicity may have an effect on the significant associations found, and their direction. Future research questions to be explored include whether higher klotho levels are always associated with better outcomes, across the lifespan, ethnic groups, sex, and across various health metrics. Yokoyama et al. (2017) found that in late life, *KL-*VS heterozygotes had higher klotho serum levels, and homozygotes had lower klotho levels, relative to non-carriers. However, it is unclear whether the relationship between *KL-*VS genotype and klotho levels remains constant during the life course. Furthermore, brain development is a noisy process. Longitudinal data might uncover meaningful associations, which cross-sectional data might miss. A fuller understanding of the precise mechanisms by which *KL-*VS genotype and klotho protein levels affect the brain, health and survival could lead to strategies that promote both early-life development and late-life healthy aging.

## Electronic supplementary material

Supplementary Table 1 (DOCX 15 kb)
