# Supplementary material for: Klotho gene polymorphism, brain structure and cognition in early-life development
Source: Brain Imaging Behav. 2018 Nov 5;14(1):213–25. doi: 10.1007/s11682-018-9990-1 (PMC6588504; doi:10.1007/s11682-018-9990-1)
Supplement: Supplementary file 1 — (DOCX 15 kb) [file 11682_2018_9990_MOESM1_ESM.docx]

**Supplementary Table 1 Generation R estimated coefficients of the general linear models for total grey/white/brain volume**

|  | Total Brain  Volume | | Total Grey Matter Volume | | | | | Total White Matter volume | | | |  |  |
| --- | --- | --- | --- | --- | --- | --- | --- | --- | --- | --- | --- | --- | --- |
|  | *B* | *p* | *B* | | *p* | | | *B* | | *p* | |  |  |
| Model 1 - KL-VS, main effect | 0.003 (0.004) | .515 | 0.001 (0.001) | | .503 | | | 0.001 (0.002) | | .739 | |  |  |
|  |  |  |  |  | |  | | |  | |  | | |
| Model 2 - KL-VS x Age | 0.005 (0.003) | .130 | 0.002 (0.001) | | .179 | | | 0.001 (0.001) | | .234 | |  |  |
|  |  |  |  | | | |  |  | | |  | |  |
| Model 3 - KL-VS x Sex | -0.009 (0.009) | .319 | 2×10^-4^ (0.003) | | .934 | | | -0.005 (0.003) | | .151 | |  |  |

*B indicates the estimated coefficients, p indicates the corresponding p-values. The numbers inside the brackets indicate the standard errors. N = 2306. Three separate models were run. Model 1 tests whether* KL-VS *has a main effect. Models 2 and 3 test the two-way interaction effects of age-by-*klotho *and sex-by-*klotho, respectively*. All models were adjusted for age at MRI, age^2^, sex, total intracranial volume, genetic ancestral background (first 7 principle components), and MRI scanner.*
